# Association between GRIN2B DNA methylation and cognitive impairment: a cross-sectional study of patients with bipolar depression

**DOI:** 10.3389/fpsyt.2025.1574391

**Published:** 2025-05-14

**Authors:** Hao Yu, Chengji Wang, Yao Wu, Changxing He, Shaohong Zou

**Affiliations:** Department of Clinical Psychology, People’s Hospital of Xinjiang Uygur Autonomous Region, Urumqi, China

**Keywords:** bipolar disorder, depression, DNA methylation, GRIN2B, cognitive impairment

## Abstract

**Background:**

Cognitive impairment is a prevalent feature throughout the course of bipolar disorder (BD) and may contribute to recurrent episodes and poor prognosis. Despite its significant clinical impact, the biological mechanisms underlying cognitive impairment in BD remain poorly understood, complicating treatment efforts. The NR2B subunit of the N-methyl-D-aspartate (NMDA) receptor, encoded by the GRIN2B gene, plays a critical role in cognitive functions.

**Methods:**

In this study, we measured the methylation levels of the promoter region of the GRIN2B gene in peripheral blood samples from patients with bipolar depression and healthy controls using the MassARRAY method. Cognitive performance was assessed through a series of standardized neuropsychological tests. Subsequently, we analyzed the correlation between GRIN2B gene promoter methylation levels and cognitive performance in patients with bipolar depression.

**Results:**

We identified aberrant methylation levels at multiple CpG sites within the GRIN2B gene promoter region in patients with bipolar depression compared to healthy controls. These methylation changes were significantly associated with impairments in several cognitive domains, including attention and executive function, even after adjusting for potential confounding factors. These findings suggest that aberrant methylation in the GRIN2B gene promoter region may play a critical role in cognitive impairment in bipolar depression.

**Conclusions:**

DNA methylation levels in the GRIN2B gene promoter region may represent a potential therapeutic target for addressing cognitive impairment in bipolar depression. These findings provide a theoretical foundation for future clinical diagnosis and the development of targeted treatment strategies.

## 1 Introduction

Bipolar disorder (BD) is a complex and severe mental health condition that affects over 70 million individuals globally (1). Historically, BD has been recognized for its significant impact on global health, ranking as the sixth highest cause of disability worldwide as reported by (2). The depressive phase of BD is particularly concerning, as it often marks the onset of the disorder and is associated with substantial psychosocial impairment and an elevated risk of suicide (3). Cognitive impairment is regarded as a core feature of bipolar disorder, with research indicating that it persists throughout the disorder’s course, including during periods of remission (4),t his cognitive dysfunction spans multiple domains, impacting areas such as attention, memory, and executive function (5, 6). Moreover, cognitive impairment may hinder emotional regulation in individuals experiencing bipolar depression, potentially leading to recurrent episodes and contributing to a poor prognosis. Such impairments exacerbate the overall disease burden and negatively affect the quality of life for those affected (7, 8). Therefore, addressing cognitive impairment in patients with BD, particularly those with bipolar depression, is crucial. However, the mechanisms underlying cognitive impairment in BD patients remain poorly understood.

The N-methyl-D-aspartic acid receptor (NMDAR) plays a critical role in synaptic plasticity and memory formation. Notably, overexpression of the GRIN2B gene, which encodes the NMDAR 2B subunit, has been shown to enhance synaptic function and improve cognitive performance (9, 10). Recent studies have demonstrated that patients with bipolar disorder exhibit reduced NMDAR activity in key brain regions, such as the dorsolateral prefrontal cortex, anterior cingulate gyrus, and hippocampus, suggesting that the GRIN2B gene may play a pivotal role in the pathophysiology of BD (11). Furthermore, genetic association studies in the Chinese Han population have identified a significant positive correlation between specific single nucleotide polymorphisms (SNPs) in the GRIN2B gene and bipolar disorder (12, 13). However, it should be noted that mRNA levels of GRIN2B were not significantly associated with BD in these studies.

The interaction between genetic and environmental factors is a major contributor to the pathogenesis of BD (14). In fact, environmental factors can be as influential as genetic inheritance in the development of BD, with childhood stress being a primary source of environmental influence (15, 16). Epigenetics, which represents the interplay between genetic and environmental factors, has been implicated in the pathophysiology of bipolar disorder (17–19). Among various epigenetic mechanisms, DNA methylation is considered the most stable and significant mode of epigenetic regulation in BD (20, 21). This process primarily occurs in the promoter regions of genes (22).

Previous studies have found that cognitive deficits in rats exposed to subchronic phencyclidine stimulation are associated with increased DNA methylation levels at the promoter region of the GRIN2B gene in the prefrontal cortex and hippocampus (23). Similarly, cognitive decline in patients with schizophrenia has been linked to altered methylation levels in the GRIN2B gene promoter (24). However, current research on GRIN2B gene methylation and cognitive function has primarily focused on animal models. Given this context, we hypothesize that changes in DNA methylation levels in the promoter region of the GRIN2B gene in patients with bipolar depression may lead to reduced expression of NMDAR receptor subunits and glutamatergic dysfunction, thereby affecting cognitive function. To test this hypothesis, we compared the cognitive functions of patients with bipolar depression and healthy controls while measuring DNA methylation levels in the promoter region of the GRIN2B gene in peripheral blood. We further correlated these changes with cognitive functions and other clinical data.

## 2 Methods

### 2.1 Participants

The sample size was calculated using G*Power (version 3.1.9.7) based on a correlation analysis between GRIN2B promoter DNA methylation and cognitive function, with an expected correlation coefficient (r) of 0.45 (24). With a significance level (α) of 0.05 (two-tailed) and 80% power (1−β), the required sample size was 39 participants per group (25). Due to recruitment challenges and strict inclusion/exclusion criteria, the final sample included 31 patients with bipolar depression cognitive impairment and 39 healthy controls. *Post-hoc* power analysis indicated 75% power for detecting the expected correlation in the case group. Rigorous quality control were performed to ensure the robustness of the findings.

The patients with bipolar depression were recruited from attending the outpatient and inpatient clinics of the Department of Clinical Psychology at the People’s Hospital of Xinjiang Autonomous Region from April 2023 to December 2023. The inclusion criteria were: (1) Meeting the Diagnostic and Statistical Manual of Mental Disorders, Fifth Edition (DSM-5) criteria for bipolar disorder; (2) A definitive diagnosis confirmed by two senior-level psychiatrists; (3) Age between 18 and 55 years, without gender restrictions; (4) A total score above 20 on the 24-item Hamilton Depression Scale (HAMD-24) and a score below 7 on the Young Mania Rating Scale (YMRS); (5) A lower secondary school education level or above, with the ability to complete a cognitive functioning assessment. Exclusion criteria were: (1) A history of serious or chronic physical illnesses; (2) Intellectual disability; (3) Other mental disorders; (4) Presence of drug, alcohol, or other psychoactive substance abuse or dependence; (5) Pregnant or breastfeeding women; To minimize the potential impact of pre-admission treatments on GRIN2B gene methylation and cognitive functioning in bipolar depressed patients, we excluded individuals who had received electroconvulsive therapy, psychiatric medication, or systemic psychotherapy in the past six months. Additionally, venous blood samples were collected from all patients upon admission, during which cognitive function was assessed and refined. A total of 31 patients with bipolar depression, meeting the above criteria, were included in the study as the bipolar depression group, referred to as BDP.

Hospital staff and students from the Medical University were recruited as the control group during the same period and were required to fulfill the following criteria. The inclusion criteria were: (1) No mental illness or family history, as assessed by a Senior psychiatrist; (2) At least a lower secondary school education or above, and able to complete a cognitive functioning assessment; (3) An age range between 18 years old and 55 years old, with no gender restrictions. The exclusion criteria were the same as for the BDP group. A total of 39 healthy controls were included in the study as healthy controls (HC). This study was approved by the Ethics Committee of the Xinjiang Uygur Autonomous Region People’s Hospital (Ethical lot number: KY2023020968). All subjects volunteered to participate in this study and signed an informed consent form either by themselves or through their legal guardians.

### 2.2 Sociodemographic and clinical assessment

General information was collected from both groups using a self-designed questionnaire, which included age, sex, Chinese ethnicity, years of education, smoking and drinking history, and Body Mass Index (BMI). The duration of illness and age of onset were additionally recorded in patients with bipolar depression, and their clinical symptoms were assessed and scored using the Hamilton Depression Scale (HAMD-24) and the Young Mania Rating Scale (YMRS). These scales are widely used in patients with bipolar disorder and have demonstrated good reliability and validity (26, 27). All researchers were trained to ensure assessment consistency.

### 2.3 Cognitive evaluation

We used a series of neuropsychological tests to assess various dimensions of cognitive function.(1)Montreal cognitive assessment(MoCA):This scale includes cognitive function dimensions such as visuospatial and executive functions, naming, language, abstraction, memory, attention, orientation, etc. The scores for each dimension are summed to give a total score of 30 points (28); (2)Trail Making Test A(TMT-A):This scale is Used to reflect attention and information processing speed, with results based on the total completion time (29–31); (3)Digit Span Test (DST):This scale is designed to assess short-term memory and attention (32), consisting of two parts: Digit Span Forward (DS-F), Digit Span Backward (DS-B);(4)Stroop Color and Word Test (SCWT):This test is divided into three parts, each assessing different aspects of the subject’s executive function. The number of correct readings in each of the three parts is recorded, with better executive function represented by higher accuracy in the color-word section (33, 34). All participants underwent cognitive function assessments conducted by uniformly trained psychiatrists in a quiet, standardized psychometric room before the initiation of any new treatment.

### 2.4 DNA methylation detection

**Figure 1:**
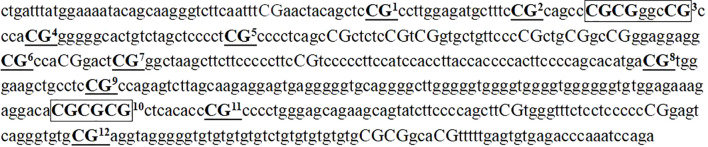
Detected sequences and analyzed 12 CpG sites in the promoter region of the GRIN2B gene.

The MassARRAY technique (35) was used to assay the methylation level of CpG sites in the promoter region of the GRIN2B gene. (1) Approximately 5 mL of peripheral venous blood was drawn into EDTA anticoagulant tubes (BD, USA) within 24 hours of hospital admission, prior to the initiation of any new pharmacological treatments. This approach was implemented to minimize the potential confounding effects of psychotropic medications on DNA methylation patterns. The whole genome DNA was extracted using a solution-based DNA extraction kit (Wuhan Genenode Biotech, China). The extracted DNA was then stored at -80°C after quality control. (2) We used the Agena EpiDesigner program (http://www.epidesigner.com) to design the primer scheme for the target sequence, which was synthesized by Xinjiang Ouyi Biotech in China. The forward primer sequence was 5’-aggaagagagagTTGATTTATGGAAAATATAGTAAGGGT T-3’, and the reverse was 5’-cagtaatacgactcactatagggagaaggctTCTAAATTTAAATCTCACACTCAAAA A-3’. The products of the primer sequence PCR pre-test were successfully detected by electrophoresis and subsequently analyzed in a DNA methylation assay.(3) The DNA was modified and purified with sodium bisulfite using the EZ DNA Methylation-Gold™ Kit (Zymo Research, USA), enriched and amplified by PCR, followed by shrimp alkaline phosphatase digestion, transcriptional cleavage, and resin purification. The resin-purified products were then transferred to a 384-well SpectroCHIP^®^ bioarray (Axygen, USA) by the Agena Nanodispenser RS1000 Spotting Instrument (Axygen, USA) for spotting. The spotted SpectroCHIP chips were further analyzed by matrix-assisted laser desorption/ionization time-of-flight mass spectrometry (MALDI-TOF-MS) using a MassARRAY Analyzer 4.0 mass spectrometer (Axygen, USA) to produce mass spectra. Finally, the mass spectrometry methylation levels were obtained by Epityper 1.2 software (Sequenom, USA). A total of 12 CpG sites in the promoter region of the GRIN2B gene located at 1259–1755 bp (497 bp in length) were detected for methylation levels (**Figure 1**).

### 2.5 Statistical analysis

The Statistical Package for the Social Sciences (SPSS) version 27.0 and Origin version 8.5.0 (Pro 2024) were used for data analysis and visualization. The normality of the data was checked using the one-sample Shapiro-Wilk test. Comparisons between the two groups were made using the Student t-test for data that met normality assumptions, and the Mann-Whitney U-test for data that did not. Categorical data were analyzed using the chi-square test or Fisher’s exact test. Spearman correlation were used to examine the correlation between cognitive impairment and the methylation level of each CpG site in the promoter region of the GRIN2B gene in patients with bipolar depression, and further performed a partial correlation analysis controlling for potential confounders such as age, age of onset, duration of disease and years of education. To account for multiple testing in the correlation analysis, the False Discovery Rate (FDR) correction was applied using the Benjamini-Hochberg procedure, with an FDR threshold set at Q=0.05. We note that the BDP group was categorized into mild cognitive impairment(MCI) and severe cognitive impairment(SCI) groups based on the MoCA score (36, 37). Subsequently, a one-way logistic regression analysis was performed with the mild and moderate-to-severe groups as the dependent variables, and the methylation rate of each GRIN2B gene locus, as well as general and clinical information, were used as covariates. Variables with P<0.1 were then entered into multifactorial logistic regression to explore the influencing factors of cognitive impairment severity in bipolar depression patients. The significance level was set at α=0.05, with two-sided tests.

## 3 Results

### 3.1 Demographic and clinical assessment

A total of 31 bipolar depression patients (23 women) and 39 healthy volunteers (27 women) were included in this study. For the bipolar depression patients, the median (interquartile range) values for the duration of illness, age at onset, HAMD-24 score, and YMRS score were 4 (1.42, 8),20 (16.58, 31),32 (23, 36), and 2 (2, 3), respectively. There were no statistically significant differences between the two groups when comparing age, gender, education, BMI, smoking, or drinking (**Table 1**, P>0.05).

**Table 1:** Demographic and clinical comparisons of BDP and HC.

Variable	BDP(n=31)	HC(n=39)	χ2/t/Z	p
Age (years)	23 (20, 39)	26 (22, 35)	1.345	0.179
Education (years)	16 (15, 16)	16 (16, 16)	0.604	0.546
BMI (kg/m²)	23.65 ± 0.75	23.17 ± 0.52	0.538	0.592
Gender (male/female)	(8,23)	(12,27)	0.208	0.648
Smoking (Y,N)	(6,25)	(8,31)	0.014	0.904
Drinking (Y,N)	(6,25)	(7,32)	0.023	0.881
Age of Onset	20 (16.58, 31)	–	–	–
Disease Duration (years)	4 (1.42, 8)	–	–	–
HAMD-24	32 (23, 36)	–	–	–
YMRS	2 (2, 3)	–	–	–

BDP, bipolar depression; HAMD-24,24-item Hamilton Depression Scale; YMRS, Young Mania Rating Scale; Y,N, Yes or No.

### 3.2 Comparisons of cognitive functions and GRIN2B DNA methylation level between BDP and HC

#### 3.2.1 Cognitive functions

In **Table 2**, the BDP group had significantly lower total MoCA scores than the healthy controls (U=6.799, P<0.001), and their performance on the MoCA subscales was also inferior to that of the HC group (P<0.001). In the SCWT, the BDP group showed lower scores than the HC group in Word-reading (U=3.577, P<0.001), Color-naming (t=-3.815, P<0.001), and Color-word (t=-4.535, P<0.001). Performance on DSF (U=3.866, P<0.001) and DSB (U=2.715, P<0.05) in the BDP group was similarly lower than in the HC group. The TMT-A completion time in the BDP group was longer than in the healthy population (U=-4.901, P<0.001).

**Table 2:** Comparison of cognitive function between BDP and HC.

Variable		BDP (n=31)	HC (n=39)	*t/U*	Raw p-value	Adjusted p-value (BH)
MoCA (scores)	Total Score	21 (17,24)	28 (27,30)	6.799	<0.001	<0.001
	Visual space and Executive function	2 (2,3)	5 (4,5)	6.531	<0.001	<0.001
	Name ability	3 (2,3)	3 (3,3)	3.734	<0.001	<0.001
	Delayed memory	3 (1.5,4)	4 (4,5)	4.649	<0.001	<0.001
	Attention	4 (4,5.5)	6 (6,6)	5.161	<0.001	<0.001
	Language ability	1 (1,2)	2 (2,3)	4.741	<0.001	<0.001
	Abstract ability	1 (1,2)	2 (2,2)	4.718	<0.001	<0.001
	Directional force	5 (5,5.5)	6 (6,6)	6.408	<0.001	<0.001
SCWT (scores)	Word-reading	82 (62,96)	98 (91,100)	3.557	<0.001	<0.001
	Color-naming	55.97 ± 3.63	71.67 ± 1.94	-3.815	<0.001	<0.001
	Color-word	33.42 ± 2.45	46.62 ± 1.72	-4.535	<0.001	<0.001
DST (scores)	Forward	10 (8,12)	12 (11,13)	3.866	<0.001	<0.001
	Backward	6 (5,7.5)	8 (6,9)	2.715	0.007	0.012
TMT (seconds)	Part-A	46.45 (36.98,71.75)	26.55 (23.39,36.46)	-4.901	<0.001	<0.001

BDP, bipolar depression; MoCA, Montreal cognitive assessment; SCWT, Stroop Color and Word Test; DST, Digit Span Test; TMT, Trail Making Test. Raw p-value, The unadjusted p-value. Adjusted p-value (BH): The p-value adjusted for multiple testing using the Benjamini-Hochberg procedure to control the False Discovery Rate (FDR).

#### 3.2.2 GRIN2B DNA methylation level

In this study, a total of 12 CpG sites within the CpG island of the GRIN2B promoter region were examined for their methylation levels. Differential methylation sites between the BDP and HC groups were identified at seven CpG sites, specifically CpG1, CpG3, CpG5, CpG7, CpG9, CpG10, and CpG12. Among these sites, the differences in methylation levels at CpG3, CpG7, and CpG10 were particularly prominent, with statistically significant results (p<0.001). In contrast, no statistically significant differences were observed in the methylation levels of the other sites between the two groups (P>0.05, **Table 3**). Further analysis revealed that the methylation levels of CpG1 and CpG7 in the BDP group were lower than those in the HC group (p<0.05). In contrast, the methylation levels of CpG3, CpG5, CpG9, CpG10, and CpG12 in the BDP group were higher than those in the HC group (p<0.05).

**Table 3:** Comparison of methylation rates of various CpG sites in the GRIN2B gene promoter region between BDP and HC.

Methylation % at CpG site	BDP (n=31)	HC (n=39)	t	Raw p-value	Adjusted p-value (BH)
CpG1 (%)	31.59 ± 13.28	39.12 ± 11.59	-2.532	0.014	0.025
CpG2 (%)	43.72 ± 16.02	46.42 ± 11.31	-0.795	0.430	0.573
CpG3 (%)	52.51 ± 10.00	28.09 ± 28.44	4.990	<0.001	<0.001
CpG4 (%)	51.47 ± 12.90	52.78 ± 7.30	-0.505	0.616	0.704
CpG5 (%)	44.25 ± 10.85	36.58 ± 14.45	2.456	0.017	0.029
CpG6 (%)	41.05 ± 12.74	38.47 ± 10.91	0.913	0.364	0.506
CpG7 (%)	39.60 ± 10.89	51.09 ± 12.40	-4.061	<0.001	<0.001
CpG8 (%)	33.58 ± 12.32	32.12 ± 20.51	0.371	0.712	0.759
CpG9 (%)	44.05 ± 12.30	37.72 ± 11.16	2.254	0.027	0.041
CpG10 (%)	36.03 ± 13.94	19.12 ± 13.02	5.232	<0.001	<0.001
CpG11 (%)	40.02 ± 12.83	42.31 ± 12.60	-0.751	0.455	0.582
CpG12 (%)	43.44 ± 19.28	33.05 ± 16.23	2.446	0.017	0.027

BDP, bipolar depression. Raw p-value, The unadjusted p-value. Adjusted p-value (BH): The p-value adjusted for multiple testing using the Benjamini-Hochberg procedure to control the False Discovery Rate (FDR).

### 3.3 Cognitive test performance in relation to GRIN2B methylation levels and portion of demographic and clinical variables in BDP group

**Figure 2:**
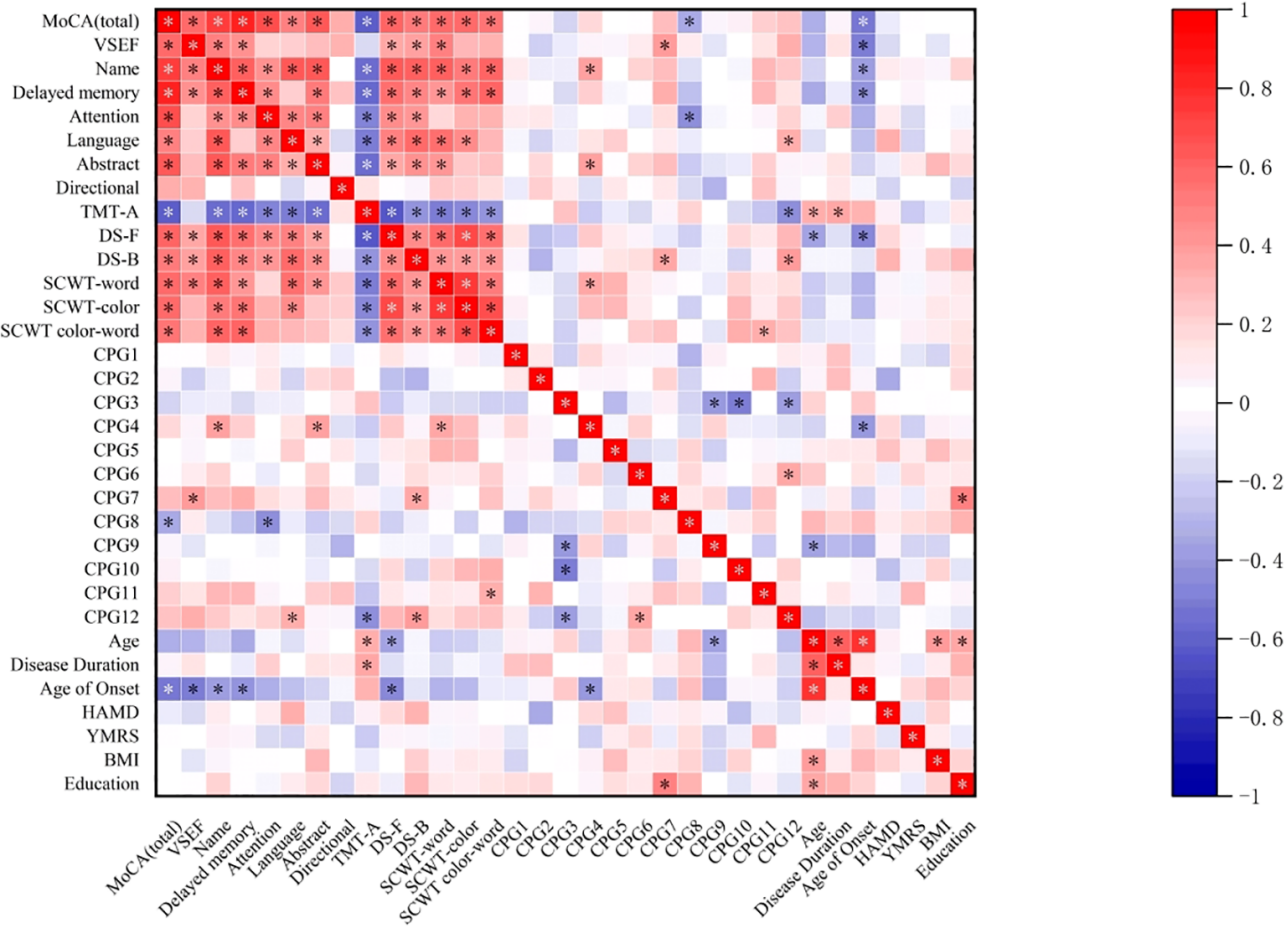
Correlation heatmap depicting the relationship between cognitive test performance and GRIN2B methylation levels and the portion of demographic and clinical variables in the BDP group. MoCA, Montreal cognitive assessment; VSEF, Visual space and Executive Function; TMT-A, Trail Making Test part-A;DS-F, Digit Span Forward; DS-B, Digit Span Backward; SCWT, Stroop Color and Word Test; HAMD, Hamilton Depression Scale; YMRS, Young Mania Rating Scale; BMI, body mass index (kg/m^2^). * *p<0.05*.

In the correlation heat map (**Figure 2**), the level of CpG4 methylation at sites in the promoter region of the GRIN2B gene was positively correlated with naming (r=0.389, P=0.031) and abstraction scores (*r*=0.397, *p*=0.027) on the MoCA subscales, as well as with word scores on the SCWT (*r*=0.363, *p*=0.045). Additionally, the level of CpG7 methylation was positively correlated with visuospatial and executive function scores on the MoCA (*r*=0.409, *p*=0.022),DS-B scores (*r*=0.372, *p*=0.040) and years of education(*r*=0.508, *p*=0.004).Interestingly, we also observed that the methylation level of CpG11 was positively correlated with color-word scores on the SCWT (r=0.358, P=0.048). In contrast, the level of CpG8 methylation was negatively correlated with total MoCA scores (r=-0.351, P=0.049) and attention scores on the MoCA (r=-0.449, P=0.011). Moreover, we found that the methylation level of CpG12 was positively correlated with language scores on the MoCA (*r*=0.357, *p*=0.048) and DS-B scores (*r*=0.412, *p*=0.021), while it was negatively correlated with the time required for TMT-A completion (*r*=-0.449, *p*=0.011). Age also demonstrated significant correlations: it was negatively correlated with the DS-F score (r=-0.357, P=0.049) and positively correlated with the time required for TMT-A (r=0.376, P=0.037). Additionally, the age of onset was negatively correlated with the total MoCA score, its subscale scores, and the DS-F score (P<0.05). The duration of disease was positively correlated with the time required for TMT-A (*r*=0.366, *p*=0.043). However, no significant association was observed between the remaining clinical variables, particularly HAMD scores, and either CpG locus or cognitive function, as the statistical p-values were greater than 0.05.To further understand the relationship between the methylation level of the GRIN2B gene and cognitive impairment, we conducted a bias-corrected correlation analysis, controlling for confounders such as age, age of onset, duration of disease and years of education. This analysis revealed that the methylation level of the CpG2 locus was negatively correlated with Visual space and executive function scores (r=-0.380, P=0.046) and DS-B scores (*r*=-0.427, *p*=0.024). Similarly, the methylation level of CpG8 was negatively associated with attention scores (*r*=-0.462, *p*=0.013).Conversely, the methylation level of CpG4 was positively correlated with naming scores *(r*=0.417, *p*=0.027), and the methylation level of CpG6 was positively correlated with SCWT color-word scores (*r*=0.425, *p*=0.024).

### 3.4 Binary logistic regression analysis based on severity of cognitive impairment in BDP group

To further explore the relationship between the severity of cognitive impairment and demographic, clinical information, and methylation levels at various sites in the promoter region of the GRIN2B gene in the BDP group, we conducted binary logistic regression analyses, beginning with univariate analysis followed by multivariate analysis. The BDP group was divided into a mild cognitive impairment group (n=21) and severe cognitive impairment group (n=10, MoCA score<18) based on the MoCA total score, as previously mentioned. The severity of cognitive impairment was used as the dependent variable (Assignment: SCI=1, MCI=0). Demographic, clinical information, and methylation levels at various sites in the promoter region of the GRIN2B gene were considered as independent variables. Gender was assigned (male=1, female=0), smoking history was assigned (history of smoking=1, no history of smoking=0), and drinking history was assigned (history of drinking=1, no history of drinking=0), while the remaining indicators were measured as continuous variables. After univariate logistic regression analysis, it was found that age of onset (OR=1.144, 95% CI:1.032-1.268, *p*=0.01), age (OR=1.116,95% CI:1.030-1.210, *p*=0.008), and the methylation level of CpG11 (OR=0.921,95% CI:0.850-0.998, *p*=0.045) were associated with the severity of cognitive impairment in the BDP group (*p*<0.05). Additionally, the P-value for the methylation level of CpG7 was less than 0.1, so these variables were entered into the multivariate regression model. The other independent variables were not significant (P>0.05). Multifactorial logistic regression analysis revealed that older age (OR=1.207, 95% CI: 1.035–1.407, P=0.017) and hypomethylation of CpG11 (OR=0.861, 95% CI: 0.755–0.983, p=0.027) were associated with the severity of cognitive impairment in the BDP group, as presented in **Table 4**.

**Table 4:** Binary logistic regression analysis on degree of cognitive impairment in patients of BDP.

Variable	Single factor Logistic regression		Stepwise multivariate Logistic regression	
	p	OR (95%CI)	p	OR (95%CI)
CpG1 (%)	0.951	1.002 (0.946-1.061)	/	/
CpG2 (%)	0.610	1.013 (0.965-1.062)	/	/
CpG3 (%)	0.673	1.017 (0.941-1.099)	/	/
CpG4 (%)	0.347	0.971 (0.914-1.032)	/	/
CpG5 (%)	0.757	1.011 (0.942-1.086)	/	/
CpG6 (%)	0.856	1.006 (0.947-1.068)	/	/
CpG7 (%)	0.082**^#^**	0.929 (0.855-1.009)	0.221	0.937 (0.845-1.040)
CpG8 (%)	0.241	1.038 (0.975-1.105)	/	/
CpG9 (%)	0.226	1.042 (0.975-1.113)	/	/
CpG10 (%)	0.930	1.002 (0.949-1.059)	/	/
CpG11 (%)	0.045*	0.921 (0.850-0.998)	0.027*	0.861 (0.755-0.983)
CpG12 (%)	0.182	0.970 (0.927-1.015)	/	/
Age (years)	0.008*	1.116 (1.030-1.210)	0.017*	1.207 (1.035-1.407)
Education (years)	0.544	0.879 (0.581-1.331)	/	/
Age of Onset	0.010*	1.144 (1.032-1.268)	0.133	1.233 (0.939-1.619)
Disease Duration (years)	0.234	1.110 (0.935-1.317)	/	/
HAMD-24	0.826	1.012 (0.908-1.129)	/	/
YMRS	0.697	1.167 (0.536-2.542)	/	/
BMI (kg/m2)	0.910	1.011 (0.841-1.214)	/	/
Gender	0.612	1.600 (0.260-9.834)	/	/
Smoking	0.950	1.062 (0.160-7.061)	/	/
Drinking	0.950	1.062 (0.160-7.061)	/	/

HAMD-24, 24-items Hamilton Depression Scale; YMRS, Young Mania Rating Scale; BMI, body mass index (kg/m^2^).

#p<0.01.

*p<0.05.

## 4 Discussion

The following main findings were derived from this case-control study:(1) Patients with bipolar depression exhibited significantly more widespread cognitive impairment compared to healthy controls, particularly in domains such as memory, attention, information processing speed, and executive function.(2) Differences in DNA methylation levels in the promoter region of the GRIN2B gene were observed between the bipolar depression (BDP) and healthy control (HC) groups, and these methylation levels were associated with cognitive impairment in patients with bipolar depression.(3) Older age and higher DNA methylation levels at specific sites in the promoter region of the GRIN2B gene were identified as independent risk factors for the severity of cognitive impairment in these patients.

Our study employed the MoCA, DST, TMT-A and SCWT to evaluate cognitive function in patients with bipolar depression. The results revealed significant impairments across all cognitive domains—including memory, attention, information processing speed, and executive functioning—compared to healthy controls. While cognitive dysfunction is a well-established feature of depressive states, some findings provide further evidence that these deficits are more pronounced in bipolar depression than in unipolar depression (UD), particularly in language abilities among patients with MoCA scores below 23 (38). Importantly, our study highlights that cognitive impairments in bipolar depression extend beyond state-related deficits during depressive episodes. For instance, prior research has demonstrated that euthymic BD patients also exhibit persistent deficits in language abilities, attention, immediate memory, and executive functioning, particularly in sensitivity to interference and inhibitory control, compared to healthy controls (39, 40). Furthermore, patients with bipolar disorder in remission continue to display cognitive impairments on the TMT, DST, and SCWT, suggesting that these deficits may reflect trait-related vulnerabilities (41). Collectively, these findings underscore the dual nature of cognitive dysfunction in bipolar disorder, encompassing both state-related impairments during acute episodes and trait-related deficits that persist across mood states. This distinction is critical for understanding the longitudinal course of bipolar disorder and for developing targeted interventions. Therefore, cognitive function should be regarded as a core therapeutic target (42) in bipolar depression, and clinicians are encouraged to integrate cognitive assessment and management into standard treatment protocols to address both state- and trait-related cognitive deficits.

A previous animal study demonstrated that reduced activity of the GRIN2B subunit in the hippocampus plays a role in the induction of manic-like behavior in mice (43). Furthermore, several studies have investigated the GRIN2B gene as a candidate for understanding the pathogenesis of BD (12, 44, 45). In our research, we identified differential sites in the promoter region of the GRIN2B gene, including CpG1, CpG3, CpG5, CpG7, CpG9, CpG10, and CpG12, with varying DNA methylation levels between patients with bipolar depression and healthy controls.

Studies have suggested that the GRIN2B gene plays a significant role in cognitive impairment. Variations in GRIN2B gene expression products, such as mRNAs and proteins distributed in the hippocampus, prefrontal lobes, and related cerebral cortices, may influence cognitive functions (46–48). Certain polymorphic sites in the GRIN2B gene have been associated with deficits in memory, information processing speed, and executive function (49–51). In this study, we observed that elevated methylation levels at most sites in the promoter region of the GRIN2B gene, such as CpG1, CpG7, CpG11, and CpG12, were positively correlated with cognitive function in patients with bipolar depression, while an inverse trend was observed at the CpG8 locus. This correlation between methylation levels at specific sites and cognitive function remained significant even after controlling for potential confounders. Importantly, these findings are consistent with previous research. For instance, in patients with schizophrenia (24), an overall reduction in methylation at five CpG sites in the GRIN2B gene promoter was observed, with methylation levels at CpG4 positively correlating with cognitive outcomes. Similarly, a study (52) identified 18 CpG sites in three CpG islands surrounding the Grin2b promoter on chromosome 6 in the hippocampus of mice undergoing experimental cesarean section, all of which exhibited varying degrees of elevated DNA methylation levels associated with perioperative neurocognitive deficits in aged mice. In a separate cohort study (53), cognitive decline in boys with prenatal bisphenol F exposure was linked to hypermethylation of the CpG3 locus in the regulatory region of the GRIN2B gene. Elevated methylation levels of the GRIN2B gene may hinder the transcription process, potentially leading to decreased gene expression. Conversely, reduced methylation of GRIN2B may enable transcription factors within the general transcription factor III complex to bind and act as transcriptional repressors, resulting in the downregulation of gene expression (54–56). This regulatory mechanism appears to be dependent on the specific gene sequence.

This study found that, in addition to GRIN2B gene methylation levels, age-related factors were associated with cognitive impairment and were identified as significant risk factors for the increased severity of cognitive impairment in patients with bipolar depression. Specifically, age appears to be a strong predictor of cognitive decline in patients with bipolar disorder (BD), as evidenced by greater dysfunction in information processing speed with advancing age (57). Research suggests that BD patients experience a progressive decline in cognitive function as they age, which may be influenced by the aging process itself. Additionally, older age has been linked to selective cognitive decline, particularly in attention-related domains, among BD patients (58). Furthermore, a study (59) demonstrated that early cognitive dysfunction in first-onset BD patients was associated with significant age-related brain deterioration, as revealed by brain age estimation modeling. This early decline in cognitive function may be related to a shift in response patterns from negative to positive stimuli in BD patients as they age (60), and has been associated with reduced cortical thickness in the prefrontal lobe, cingulate gyrus, and other key brain regions, as well as diminished gray matter volumes in the hippocampus, amygdala, thalamus, and striatum in BD patients (61).

Correlation and regression analyses demonstrated that the severity of depression in patients with bipolar disorder did not significantly affect cognitive ability, the severity of cognitive impairment, or GRIN2B promoter methylation levels. This suggests that cognitive impairment in these patients may be independent of depressive symptom severity. While severe depression is often associated with dysphoria, psychomotor retardation, and sleep disturbances (e.g., lethargy or insomnia), which may confound the assessment of cognitive dysfunction by presenting it as a secondary symptom, our findings indicate that cognitive impairment in bipolar depression is not influenced by depression severity. This further implies that the relationship between GRIN2B promoter methylation levels and cognitive impairment is independent of depressive episodes. These results align with a large population-based cohort study (62), which also found no significant association between GRIN2B DNA methylation and depressive episodes in bipolar disorder. The consistency of our findings with those from a larger, well-powered study underscores the robustness of our conclusions and supports the potential role of GRIN2B methylation as an independent biomarker in bipolar disorder.

To our knowledge, this is the first study to investigate the relationship between GRIN2B promoter methylation levels and cognitive impairment in patients with bipolar depression, suggesting a potential link between the two. To minimize confounding effects, we recruited medication-naïve patients who had not received pharmacological or psychological interventions for at least six months prior to enrollment and controlled for additional variables to enhance statistical rigor. However, several limitations should be noted. First, the small sample size, constrained by assay costs and logistical challenges, may limit the robustness of our findings. Second, the use of peripheral blood as a surrogate for CNS tissue may not fully capture brain-specific methylation patterns. Third, the lack of GRIN2B gene expression data precludes exploration of potential correlations between methylation and transcriptional activity. Fourth, the relatively young mean age of our cohort (23 years), while consistent with the peak onset of mania, raises concerns about diagnostic certainty and may reflect a subgroup with earlier disease onset, potentially limiting the generalizability of our results to older populations or those with later-onset illness. Finally, the exclusion of patients in different disease phases, particularly remission, restricts the broader applicability of our findings. Future studies should address these limitations by expanding sample sizes, incorporating gene expression analyses, enrolling patients across a wider age range and disease stages, and investigating potential age- and subtype-related differences in GRIN2B methylation.

## 5 Conclusion

In conclusion, patients with bipolar depression exhibit widespread cognitive impairment and abnormal DNA methylation levels in the GRIN2B gene. Our findings suggest that altered DNA methylation levels in the promoter region of the GRIN2B gene may be associated with reduced gene expression, which could potentially exacerbate cognitive impairment in these patients. Additionally, older age appears to be a significant factor influencing the severity of cognitive impairment. This study provides a theoretical foundation for further exploration of the clinical diagnosis and treatment of cognitive impairment in bipolar depression and identifies potential therapeutic targets for future research.

## Data Availability

The raw data supporting the conclusions of this article will be made available by the authors, without undue reservation.
